# Novel variants in the RDH5 Gene in a Chinese Han family with fundus albipunctatus

**DOI:** 10.1186/s12886-022-02301-5

**Published:** 2022-02-11

**Authors:** Tianwei Qian, Qiaoyun Gong, Hangqi Shen, Caihua Li, Gao Wang, Xun Xu, Isabelle Schrauwen, Weijun Wang

**Affiliations:** 1 Department of Ophthalmology, Shanghai General Hospital, Shanghai Jiao Tong University, No. 100 Haining Rd, Shanghai, 200080 China; 2 National Clinical Research Center for Eye Diseases, Shanghai, China; 3 Shanghai Key Laboratory of Ocular Fundus Diseases, Shanghai, China; 4 Shanghai Engineering Center for Visual Science and Photomedicine, Shanghai, China; 5 Shanghai Engineering Center for Precise Diagnosis and Treatment of Eye Disease, Shanghai, China; 6 Department of Neurology, Columbia University Medical Center, 630W 168th St, New York, 10032 USA; 7 Genesky Biotechnologies Inc, Shanghai, China

**Keywords:** Fundus albipunctatus, RDH5 gene, Frameshift deletion, Missense variants

## Abstract

**Background:**

The aim of this study is to identify the genetic defects in a Chinese family with fundus albipunctatus.

**Methods:**

Complete ophthalmic examinations, including slit-lamp biomicroscopy, dilated indirect ophthalmoscopy, fundus photography, autofluorescence, swept source optical coherence tomography (SS-OCT) and full-field electroretinography (ffERG) were performed. Genomic DNA was extracted from blood samples and whole genome sequencing was performed. Variants were validated with Sanger sequencing.

**Results:**

Six members in this Chinese family, including three affected individuals and three controls, were recruited in this study. The ophthalmic examination of three recruited patients was consistent with fundus albipunctatus. Three variants, a novel frameshift deletion c.39delA [p.(Val14CysfsX47] and a haplotype of two rare missense variants, c.683G > A [p.(Arg228Gln)] along with c.710A > G [p.(Tyr237Cys], within the retinal dehydrogenase 5 (RDH5) gene were found to segregate with fundus albipunctatus in this family in an autosomal recessive matter.

**Conclusion:**

We identified novel compound heterozygous variants in RDH5 responsible for fundus albipunctatus in a large Chinese family. The results of our study further broaden the genetic defects of RDH5 associated with fundus albipunctatus.

**Supplementary Information:**

The online version contains supplementary material available at 10.1186/s12886-022-02301-5.

## Background

Fundus albipunctatus (FA; Online Mendelian Inheritance in Man identifier, OMIM #136880), a kind of autosomal recessive form disease, is mainly characterized by nonprogressive night blindness [1]. A lot of small white or pale-yellow spots are scattered in the retina and the macula may or may not be involved [1, 2]. With the increase of age, the shape and number of spots in the retina will change, or even disappear completely [2, 3]. FA was discriminated from a similar disease called retinitis punctata albescens (RPA) [4, 5], and pigmentary degeneration, narrow vasculature and visual field loss are the main characteristics of RPA different from FA. After the standard 30-min dark adaptation, standard full-field electroretinograms (ERGs) show severe reduction in rod responses, while after prolonged dark adaptation for nearly 3 h, the rod responses almost can recover to normal or near-normal levels [3, 6, 7].

The retinol dehydrogenase 5 (RDH5, OMIM 601617) gene, located on chromosome 12q13-q14 and encoding 11-cis-retinol dehydrogenase [8–10], is found in abundance in the smooth endoplasmic reticulum of the retinal pigment epithelium (RPE) [11]. This enzyme is a 32-kDa membrane-bound enzyme with 318 amino acids [12, 13]. Variants in RDH5 are associated with fundus albipunctatus and the first identification of clinically significant changes of the RDH5 sequence has been reported in 1999 [14].

In this study, we described the clinical features and molecular genetic results of a large Chinese Han family affected with FA. Novel variants in the RDH5 gene are presented and we expand the spectrum of related genetic defects associated with FA.

## Methods

### Subject recruitment and clinical examination

**Fig. 1:**
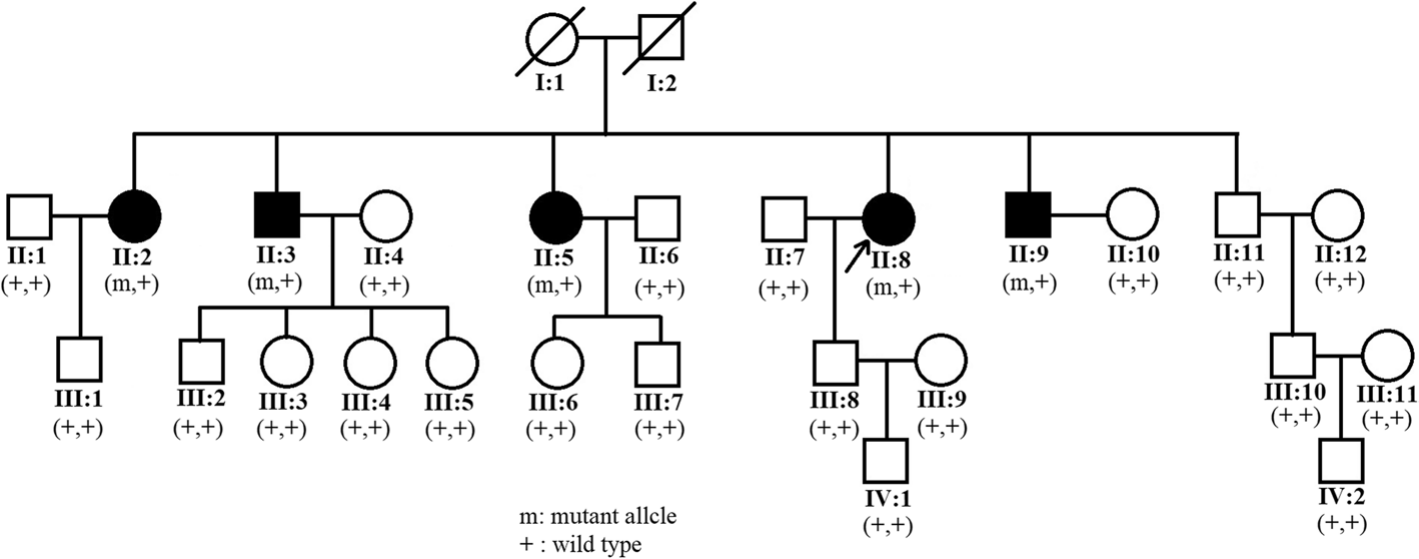
Pedigree of the family with Fundus Albipunctatus. Solid symbols indicate affected individuals, and open symbols indicate unaffected individuals. Arrow indicates the proband of this family

Six members (II:2, II:5, II:8, II:11, III:1, III:8) of the family (Fig. 1) were recruited in Shanghai General Hospital, Shanghai, China. This study was conducted in accordance with the Declaration of Helsinki and was approved by the ethics committee of Shanghai General Hospital. Informed consent was obtained from each member. Non-consanguineous marriages were found in the family. Three of the recruited six members were diagnosed with FA. A full medical history for longitudinal evaluation of the phenotype was obtained for recruited patients. Comprehensive clinical and ophthalmic examination included best corrected visual acuity, intraocular pressure measurement, slit lamp examination, dilated indirect ophthalmoscopy, fundus photography, autofluorescence, swept source optical coherence tomography (SS-OCT) and full-field electroretinography (ffERG), as well as the examination of physical malformations and neurological deficits.

### DNA preparation

Genomic DNAs were extracted from peripheral blood using the TruSeq DNA LT Sample Prep kit (Illumina, San Diego, CA) according to the manufacturer’s protocol. DNA samples were stored at − 20 °C until used, and DNA integrity was evaluated by 1% agarose gel electrophoresis [15, 16].

### Whole-genome sequencing

Whole-genome sequencing (WGS) was performed in three patients (II:2, II:5, II:8) and three unaffected family members (II:11, III:1, III:8). The libraries were constructed with the TruSeq Nano DNA LT Sample Prepararion Kit (Illumina, San Diego, CA, USA). Briefly, the genomic DNA was sheared into fragments with length ~ 350 bp using S220 Focused-ultrasonicators (Covaris, USA) [17]. Adapters were ligated onto the 3′ end of the sheared fragments. After polymerase chain reaction (PCR) amplification and purification, the final libraries were sequenced on the Illumina sequencing platform HiSeq X Ten platform (Illumina Inc., San Diego, CA, USA) and 150 bp paired-end reads were generated [18]. The raw data Q30 was above 96.3%, the average sequencing depth was at least 30× and the percentage of the genome region with coverage above 10X was 98.8%.

### Bioinformatic analysis

The raw reads were subjected to a quality check and then filtered by fastp (https://github.com/OpenGene/fastp). Reads were aligned to the human genome (hg38) using SpeedSeq [15]. Single nucleotide variants (SNV) and insertions/deletions (Indels) calling was performed using the Genome Analysis Toolkit v4. 1[19]. Structural variants (SVs) and copy number variants (CNVs) were analyzed in SpeedSeq [15]. Annotations of SNVs, InDels, SVs and CNVs were performed with ANNOVAR [20]. Variant filtering was performed as illustrated in Supplementary 1.

### Sanger sequencing

In order to verify the variants in RDH5 gene after WGS analysis, primers were designed using Primer3 software (version 4.0, http://bioinfo.ut.ee/primer3-0.4.0/). PCR primer pairs and amplification conditions are available upon request. PCR products were checked by 1% agarose gel electrophoresis and purified with SAP-Exon I kit (USB, USA) [21]. Purified PCR products were directly sequenced in both forward and reverse directions using an ABI 3730xl genetic analyzer (Applied Biosystems, Foster City, CA, USA) per manufacturer’s instructions [16, 21]. DNA sequences were analyzed using Chromas (version 2.22) and DNAMAN (version 7) software [16, 21].

## Results

### Clinical findings

**Fig. 2:**
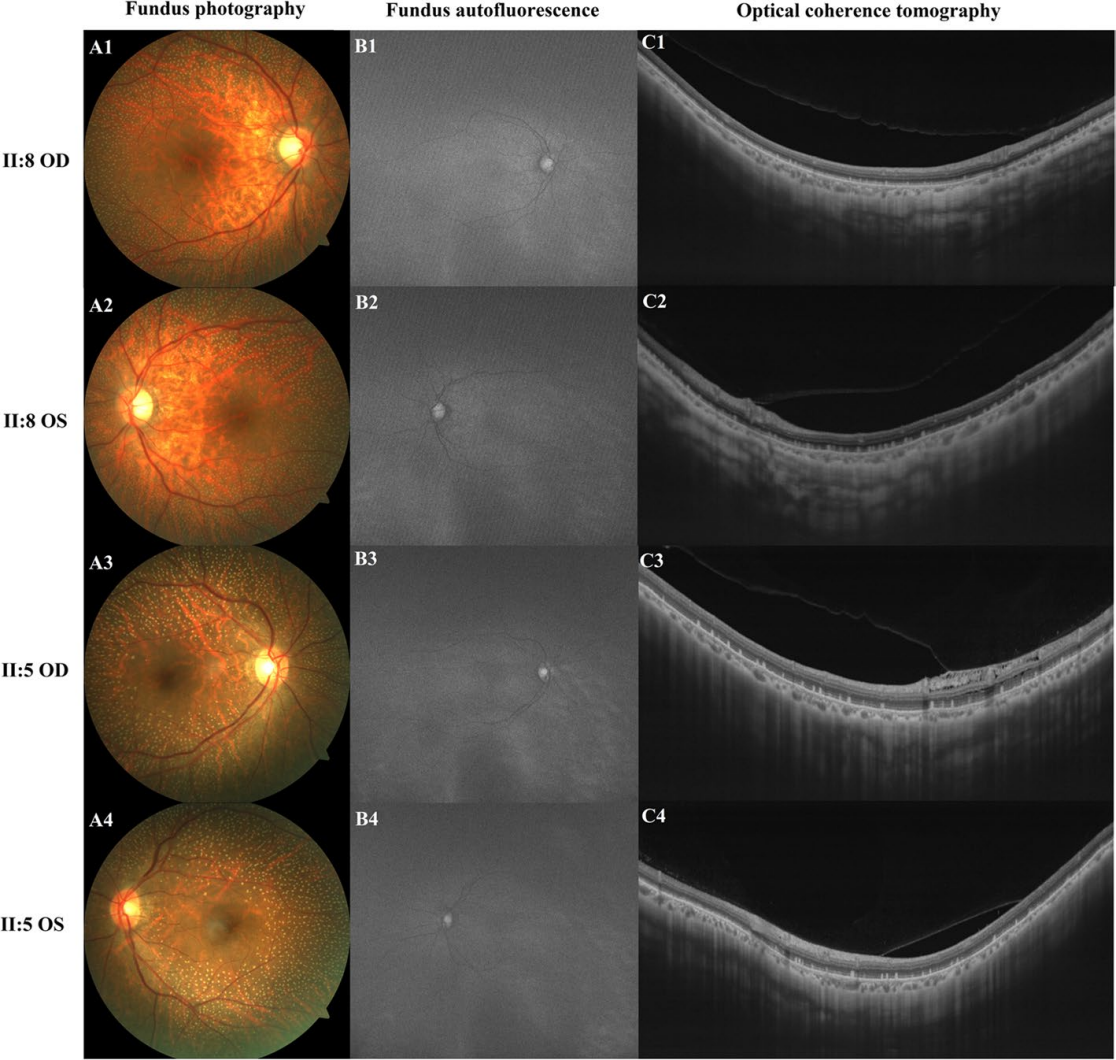
Representative ophthalmic examination results of the II:8 and II:5 patients with Fundus Albipunctatus. (A1-A4) Fundus photography. (B1-B4) Fundus autofluorescence. (C1-C4) Optical coherence tomography

The pedigree of this family is shown in Fig. 1 and suggests an autosomal recessive inheritance. The ophthalmic examination of three recruited patients within this family was consistent with fundus albipunctatus, while other three additional family members were recruited which were unaffected. As illustrated in Table 1, the three affected patients (II:2, II:5, II:8) presented night blindness in both eyes since their early childhood. They received an ophthalmic examination and showed similar clinical symptoms. Representative photos of fundus photography, autofluorescence and SS-OCT of the two patients (II:5, II:8) are shown in Fig. 2. Some non-ocular symptoms, such as intellectual disability, kidney disease, neurological deficits were not found in the patients. Three members, II:11, III:1 and III:8, have no night-blindness phenotype or other major eye diseases.

**Table 1:** Clinical characteristics and genetic variants in the RDH5 gene of the recruited individuals

Patients	Age, y	Gender	BCVA		Nucleotide Change (NM_002905.5)	Amino Acid Change (NP_002896.2)
			OD	OS		
II:2	63	F	20/60	20/40	c.39delA c.683G > A c.710A > G	p.(Val14CysfsX47) p.(Arg228Gln) p.(Tyr237Cys)
II:5	57	F	20/40	20/40	c.39delA c.683G > A c.710A > G	p.(Val14CysfsX47) p.(Arg228Gln) p.(Tyr237Cys)
II:8	54	F	20/100	20/80	c.39delA c.683G > A c.710A > G	p.(Val14CysfsX47) p.(Arg228Gln) p.(Tyr237Cys)
II:11	51	M	20/25	20/25	–	–
III:1	38	M	20/20	20/20	c.39delA	p.(Val14CysfsX47)
III:8	32	M	20/20	20/20	c.683G > A c.710A > G	p.(Arg228Gln) p.(Tyr237Cys)

*M* Male, *F* Female, *OD* The right eye, *OS* The left eye, *BCVA* Best corrected visual acuity

All patients show a moderate to severe loss of the rod-specific ERG b-wave after a standard period of dark adaptation. Table 2 shows the partial data of ffERG of the three affected patients included in this Chinese Han family.

**Table 2:** The data of ffERG of the three affected patients included in this Chinese Han family

Affected member	II:2		II:5		II:8	
	OD	OS	OD	OS	OD	OS
**ffERG, Amplitude (**μV**), dark adaption 30 min**						
**Rod response (b-wave)**						
Result	1.12	1.78	7.85	5.20	2.74	4.40
Normal range	216–341					
**Rod-cone response (a-wave)**						
Result	68.7	63.6	96.7	109	55.5	31.7
Normal range	232–375					
**Rod-cone response (b-wave)**						
Result	61.2	65.1	104	112	61	26.3
Normal range	479–568					
**Scotopic oscillatory potential (OS2)**						
Result	25.2	24.9	23.7	23.9	11.8	4.06
Normal range	77–150					
**Cone response (b-wave)**						
Result	23.3	15.1	33.5	41.5	10.8	10.9
Normal range	147–222					
**Photopic Flicker 30 Hz (N1-P1)**						
Result	31.7	23.3	48.8	65.7	11.0	33.7
Normal range	99–171					

Abbreviations: *ffERG* Full field electroretinography

### Variant analysis and verification

**Fig. 3:**
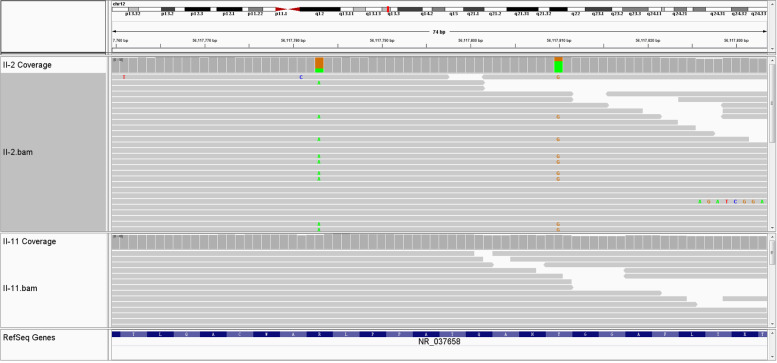
The comparison of high throughput sequencing between one affected (II:2) and one unaffected (II:11) member by Integrative Genomics Viewer (IGV)

**Fig. 4:**
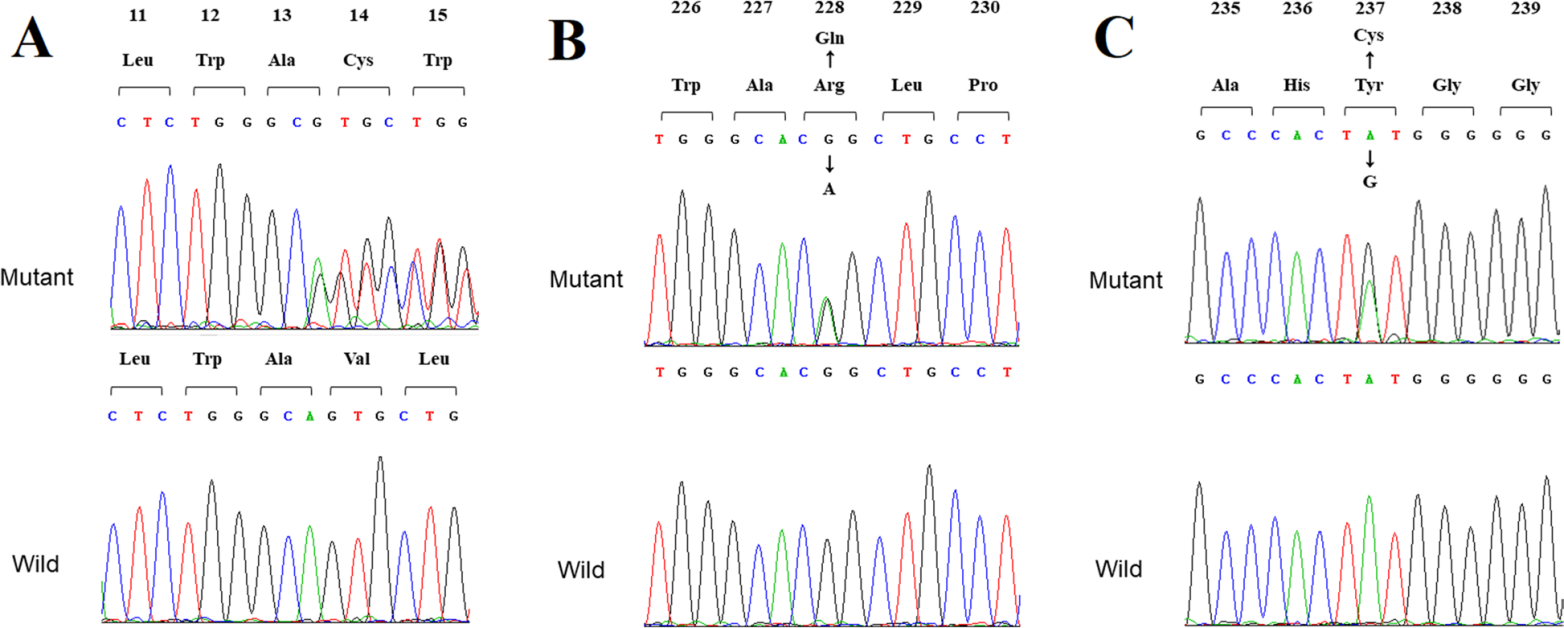
Partial Sanger traces of the RDH5 gene. The arrow indicates the variant position. (**A**) A novel frameshift deletion c.39delA;p.(Val14CysfsX47); (**B** and **C**) Missense variants c.683G > A;p.(Arg228Gln) and c.710A > G; p.(Tyr237Cys) which are present on the same haplotype

Whole genome sequence data of the affected family members was compared with that of other three unaffected family members. Three variants, a novel frameshift deletion NM_002905.5:c.39delA; p.(Val14CysfsX47) and a haplotype of two missense variants [c.683G > A; p.(Arg228Gln) and c.710A > G; p.(Tyr237Cys)], were found in a compound heterozygous state within the RDH5 gene in the three patients. The frameshift variant, p.(Val14CysfsX47) is absent from the Genome Aggregation Database (gnomAD) database and predicted to lead to an early frameshift in protein translation and likely targeted by nonsense medicated decay. The two missense variants [p.(Arg228Gln) and p.(Tyr237Cys)] are mutations linked to one chromosome according to Fig. 3. And they are both located at NAD(P)-binding domain and conserved between species (GERP: 4.88 for both variants), have a Combined Annotation Dependent Depletion (CADD) score of 16.5 and 29.4 respectively, are predicted damaging by Mutation Taster and are present in low frequency in gnomAD, with a minor allele frequency of 0.0005 and 0.00007 in the South East Asian population respectively. These three variants were subsequently confirmed via Sanger sequencing (Fig. 4). The variants were not found either in any of the unaffected members and in the 300 unrelated controls from the same ethnic background. Finally, this three variant were Classifying with likely Pathogenic of the 2015 American College of Medical Genetics and Genomics (ACMG) guidelines by Intervar [22]. More specific variant annotation details can be seen in Supplementary 2 and Supplementary 3.

## Discussion

Fundus albipunctatus (FA) is a kind of flecked retinal syndrome, which also includes benign familial fleck retina, fleck retina of Kandori, Oguchi disease, retinitis punctate albescens (RPA), and vitamin A deficiency. As a rare autosomal recessive disorder, FA is characterized by impaired dark adaptation and the widely-distributed yellow or white dot lesions in the retina [1, 2]. Patients with fundus albipunctatus have suffered from night blindness since childhood. The main clinical manifestation of them is delayed dark adaptation, which means that they are difficult to adapt to conditions from bright light to dark, usually taking 2–3 h to adapt. The flecks, between the outer limiting membrane (OLM) and the outer aspect of RPE, are widely distributed at the outer edge of the retina [6]. Affected individuals typically have normal visual acuity with severely abnormal rod ERGs, but cone ERG abnormalities, macular dysfunction, and disease progression also have been reported [1, 23–30]. These patients in our study were diagnosed as fundus albipunctatus because reduced rod b waves could be found after a short time of dark-adaptation, and then the rod b waves improved after a prolonged dark-adaptational period. They also had white punctata which closely resembled those of fundus albipunctatus.

In this family and other reported cases, FA is caused by variants of RDH5 gene. However, other two genes, retinaldehyde binding protein 1 (RLBP1) gene [31, 32] and RPE-specific protein (RPE65) gene [33], can also lead to FA. Mutations in RLBP1 gene are also associated with RPA [34–36]. Furthermore, 11-cis retinol and 11-cis retinaldehyde as its ligands, RLBP1 is expressed in the RPE cells and Müller cells [37]. As the isomerase of the visual cycle and important role in 11-cis retinal production [38], RPE65 mutations also have been associated in FA-like change [39]. Therefore, molecular evaluation of RDH5 gene plays an important role in distinguishing FA and RPA.

This study described the clinical characteristics and phenotypic variation of a Chinese Han family with newly identified compound heterozygous RDH5 variants. The RDH5, encoding the 11-cis retinol dehydrogenase, is predominantly expressed in the smooth endoplasmic reticulum of the RPE [12]. RPE cells participate in the uptake and metabolism of retinoids in the retinoid cycle and play significant roles in maintaining normal visual function [11, 40]. The 11-cis retinol dehydrogenase can oxidize 11-cis-retinol to 11-cis-retinal [41], which is then transported to adjacent photoreceptors as chromophore in rhodopsin, and in the cones under dark-adapted condition [42]. RDH5 variants resulted in a significant reduction of the stability and the activities of this enzyme [43, 44]. Table 3 summarizes some mutations in the RDH5 gene that have been reported in families with FA.

**Table 3:** A literature review of some mutations in RDH5 gene associated with fundus albipunctatus

Nucleotide Change	Amino Acid Change	Reference
c.417G > T	p.Gly139Val	[6]
c.346G > C	p.Gly116Arg	[6]
c.710A > C	p.Tyr237Ser	[6]
c.55A > G	p.Arg19Gly	[6]
c.416G > T	p.Gly139Val	[6]
c.928delCinGAAG	Leu310 to GluVal	[45]
c.500G > A	p.Arg167His	[45, 46]
c.719insG	p.Ala240Glyfs17	[45]
c.175 T > A	p.Cys59Ser	[47]
c.285G > A	p.Trp95Ter	[47]
c.124C.T	p.Arg42Cys	[46]
c.524A > T	p.Tyr175Phe	[48]
c.712G > T	p.Gly238Trp	[14]
c.832C.T	p.Arg278Ter	[42]
c.71_74delTGCC	p.Leu24Profs*36	[49]
c.160C > T	p.Arg54*	[49]
c.382G > A	p.Asp128Asn	[49–51]
c.572G > A	p.Arg191Gln	[49]
c.833G > A	p.Arg278Gln	[49]
c.95delT	p.Phe32Serfs*29	[51]
c.625C > T	p.Arg209*	[51]
c.98 T > A	p.Ile33Asn	[52]
c.103G > A	p.Gly35Ser	[25, 26]
c.319G > C	p.Gly107Arg	[25, 27, 53]
c.718dupG	p.Ala240Glyfs*19	[25]
c.394 G > A	p.Val132Met	[25]
c.839G > A	p.Arg280His	[25, 53–55]
c.469C > T	p.Arg157Trp	[43]
c.530 T > G	p.Val177Gly	[54]
c.470G > A	p.Arg157Gln	[6, 56]
c.490G > T	p.Val164Phe	[23]
c.500G > A	p.Arg167His	[2]
c.758 T > G	p.Met253Arg	[57]
c.791 T > G	p.Val264Gly	[41]
c.833G > A	p.Arg278Gln	[49]
c.801C > G	p.Cys267Trp	[58]
c.841 T > C	p.Tyr281His	[25]

According to some long-term follow-up reports, usually there is no progression in rod response in patients with this kind of night blindness, but some patients, especially the elderly, cone dystrophy is progressive [26, 28, 29]. It has been estimated that more than 30% of FA patients would be affected by cone dysfunction [6, 29, 49]. Lidén et al. suggested that cone dystrophy may be the result of RPE function impairment caused by RDH5 gene mutation, or it may be the direct result of a reduced supply of 11-cis retinal to cones [44]. The ability to complete recovery of retinal function after prolonged dark-adaptation provided a new idea that RDH5-related disease may be one of suitable candidates for gene therapy.

## Conclusion

Variants in the RDH5 gene cause autosomal recessive fundus albipunctatus, a rare form of night blindness that is characterized by a delay in the regeneration of cone and rod photopigments. The present study expands our knowledge of RDH5-related retinal dysfunction. We identified three variants in RDH5, a novel frameshift deletion Val14CysfsX47, and a haplotype of rare missense variants (Arg228Gln + Tyr237Cys), are responsible for fundus albipunctatus patients in this Chinese family. These results of our study further broaden the genetic defects of RDH5 associated with fundus albipunctatus.

## Supplementary Information

**Additional file 1:** **Supplementary 1.** The process of whole-genome sequencing and variants filtration.

**Additional file 2.** RDH5 mutation results.

**Additional file 3.** RDH5 Sequences.

## Data Availability

The datasets generated during the current study are available in the National Genomics Data Center (NGDC) repository, the accession number is HRA000883 and the persistent web link is https://ngdc.cncb.ac.cn/gsa-human/s/OXeY408H.

## Abbreviations

FA: Fundus albipunctatus
RDH5: Retinol dehydrogenase 5
RPE: Retinal pigment epithelium
RPA: Retinitis punctata albescens
BCVA: Best corrected visual acuity
OCT: Optical coherence tomography
WGS: Whole genome sequencing
PCR: Polymerase chain reaction
OCT: Optical coherence tomography
ffERG: Full-field electroretinography
