# Supplementary figures and images for: Novel variants in the RDH5 Gene in a Chinese Han family with fundus albipunctatus
Source: BMC Ophthalmol. 2022 Feb 11;22:69. doi: 10.1186/s12886-022-02301-5 (PMC8840791; doi:10.1186/s12886-022-02301-5)

**Supplementary 1.** The process of whole-genome sequencing and variants filtration


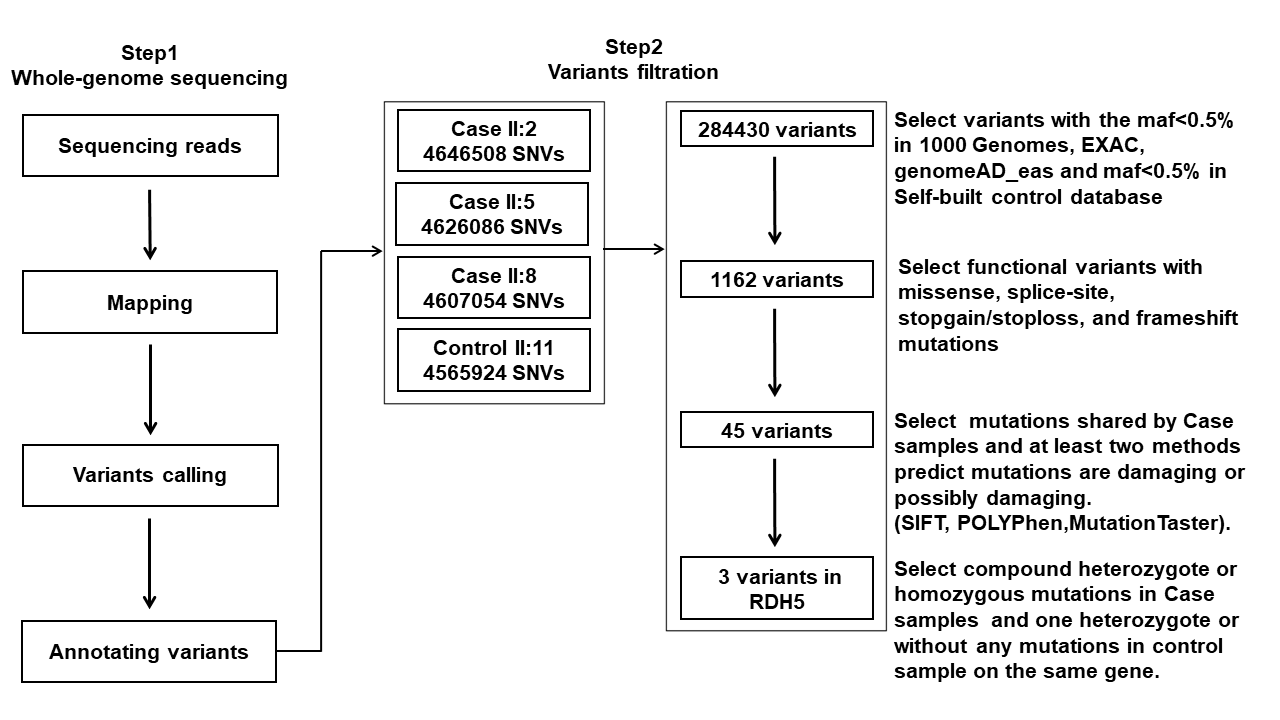

Supplement: Supplementary file 1 — Additional file 1: Supplementary 1. The process of whole-genome sequencing and variants filtration. [file 12886_2022_2301_MOESM1_ESM.docx]
