# Supplementary material for: Novel variants in the RDH5 Gene in a Chinese Han family with fundus albipunctatus
Source: BMC Ophthalmol. 2022 Feb 11;22:69. doi: 10.1186/s12886-022-02301-5 (PMC8840791; doi:10.1186/s12886-022-02301-5)
Supplement: Supplementary file 3 — Additional file 3. RDH5 Sequences. [file 12886_2022_2301_MOESM3_ESM.docx]

LOCUS NC_000012 4350 bp DNA linear CON 09-DEC-2019

DEFINITION Homo sapiens chromosome 12, GRCh38.p13 Primary Assembly.

ACCESSION NC_000012 REGION: 55720393..55724742

VERSION NC_000012.12

DBLINK BioProject: PRJNA168

Assembly: GCF_000001405.39

KEYWORDS RefSeq.

SOURCE Homo sapiens (human)

source 1..4350

/organism="Homo sapiens"

/mol_type="genomic DNA"

/db_xref="taxon:9606"

/chromosome="12"

gene 1..4350

/gene="RDH5"

/gene_synonym="9cRDH; HSD17B9; RDH1; SDR9C5"

/note="retinol dehydrogenase 5; Derived by automated

computational analysis using gene prediction method:

BestRefSeq."

/db_xref="GeneID:5959"

/db_xref="HGNC:HGNC:9940"

/db_xref="MIM:601617"

mRNA join(1..125,756..1102,1297..1555,3494..3657,3930..4350)

/gene="RDH5"

/gene_synonym="9cRDH; HSD17B9; RDH1; SDR9C5"

/product="retinol dehydrogenase 5, transcript variant 1"

/note="Derived by automated computational analysis using

gene prediction method: BestRefSeq."

/transcript_id="NM_001199771.2"

/db_xref="GeneID:5959"

/db_xref="HGNC:HGNC:9940"

/db_xref="MIM:601617"

mRNA join(1..125,761..1102,1297..1555,3494..3657,3930..4313)

/gene="RDH5"

/gene_synonym="9cRDH; HSD17B9; RDH1; SDR9C5"

/product="retinol dehydrogenase 5, transcript variant 2"

/note="Derived by automated computational analysis using

gene prediction method: BestRefSeq."

/transcript_id="NM_002905.5"

/db_xref="GeneID:5959"

/db_xref="HGNC:HGNC:9940"

/db_xref="MIM:601617"

CDS join(793..1102,1297..1555,3494..3657,3930..4153)

/gene="RDH5"

/gene_synonym="9cRDH; HSD17B9; RDH1; SDR9C5"

/note="Derived by automated computational analysis using

gene prediction method: BestRefSeq."

/codon_start=1

/product="retinol dehydrogenase 5 precursor"

/protein_id="NP_001186700.1"

/db_xref="CCDS:CCDS31829.1"

/db_xref="GeneID:5959"

/db_xref="HGNC:HGNC:9940"

/db_xref="MIM:601617"

/translation="MWLPLLLGALLWAVLWLLRDRQSLPASNAFVFITGCDSGFGRLL

ALQLDQRGFRVLASCLTPSGAEDLQRVASSRLHTTLLDITDPQSVQQAAKWVEMHVKE

AGLFGLVNNAGVAGIIGPTPWLTRDDFQRVLNVNTMGPIGVTLALLPLLQQARGRVIN

ITSVLGRLAANGGGYCVSKFGLEAFSDSLRRDVAHFGIRVSIVEPGFFRTPVTNLESL

EKTLQACWARLPPATQAHYGGAFLTKYLKMQQRIMNLICDPDLTKVSRCLEHALTARH

PRTRYSPGWDAKLLWLPASYLPASLVDAVLTWVLPKPAQAVY"

CDS join(793..1102,1297..1555,3494..3657,3930..4153)

/gene="RDH5"

/gene_synonym="9cRDH; HSD17B9; RDH1; SDR9C5"

/note="Derived by automated computational analysis using

gene prediction method: BestRefSeq."

/codon_start=1

/product="retinol dehydrogenase 5 precursor"

/protein_id="NP_002896.2"

/db_xref="CCDS:CCDS31829.1"

/db_xref="GeneID:5959"

/db_xref="HGNC:HGNC:9940"

/db_xref="MIM:601617"

/translation="MWLPLLLGALLWAVLWLLRDRQSLPASNAFVFITGCDSGFGRLL

ALQLDQRGFRVLASCLTPSGAEDLQRVASSRLHTTLLDITDPQSVQQAAKWVEMHVKE

AGLFGLVNNAGVAGIIGPTPWLTRDDFQRVLNVNTMGPIGVTLALLPLLQQARGRVIN

ITSVLGRLAANGGGYCVSKFGLEAFSDSLRRDVAHFGIRVSIVEPGFFRTPVTNLESL

EKTLQACWARLPPATQAHYGGAFLTKYLKMQQRIMNLICDPDLTKVSRCLEHALTARH

PRTRYSPGWDAKLLWLPASYLPASLVDAVLTWVLPKPAQAVY"

1 ACTCCGGACTTTGGCCTTAGCAGTAGTTAGTGTGGGAGGCTGGGAAGACTGGGAGCAGTC

61 TCTTAAACAAAAGCAAAAGAATAAGCTTCGGGCGCTGTAGTACCTGCCAGCTTTCGCCAC

121 AGGAGGTAAGTGGATACTGGGAGCTGGGGGAACTGAGAAGACTAGCCAGATATTACATGT

181 ATTGCCAACTCAAAactttcagcttttaacatgcttcctcacacattatcccctttgatc

241 ctccacaactctgaggtggacctggtgggtcttagccccacttggtagatgagaaaatag

301 gttgagagagacagtgagatgctcagtatcacacagcAAACCTCTTGGCCCTATACATCA

361 TTCCAAACACAAGACCCAGGTTGCATATAGAAGGTTCAGTGTCCCTGGTTTAGAAGGAGA

421 GGTGGTGTGAGGCAAGCAAGAagatgcctctgctgcactccagcctgggcgacagagtga

481 gactccatctcaaaaaaaaaaaaaaaaaaaaaaagaTGCCTCTGCTCCATACAGCAGGTC

541 TGTACACAGGATCTGGCTCATGTGGTTTTAGTTAAGTTAGCCACAAATACAGGGTCTGCC

601 CACATCTTTGCTTTGAACAGATGAGCCATGGTTGGCCAATTATCTGCCAACCAGATAATT

661 TCTCAATATGCTCACACCAGATGCTTCCAGCTAGGGAGGGTATTAGGGGAAAGGGCTTGA 1F

721 GGGCCACAGTAAACTGGACAAGTTTTTCTGCCCAGCCTAGGCTGCCACCTGTAGGTCACT

781 TGGGCTCCAGCT**ATG**TGGCTGCCTCTTCTGCTGGGTGCCTTACTCTGGGCAGTGCTGTGG

841 TTGCTCAGGGACCGGCAGAGCCTGCCCGCCAGCAATGCCTTTGTCTTCATCACCGGCTGT

901 GACTCAGGCTTTGGGCGCCTTCTGGCACTGCAGCTGGACCAGAGAGGCTTCCGAGTCCTG 1R

961 GCCAGCTGCCTGACCCCCTCCGGGGCCGAGGACCTGCAGCGGGTGGCCTCCTCCCGCCTC

1021 CACACCACCCTGTTGGATATCACTGATCCCCAGAGCGTCCAGCAGGCAGCCAAGTGGGTG

1081 GAGATGCACGTTAAGGAAGCAGGTAAGTATGGTAGACCACCAGGAATATGGTGTGGGGTG

1141 TCCTGATCCCCACAGTCACCCCAGGAGTCACCTGCAAGGGCTGTGGTAAGCTAAAGGGAC

1201 AATTTGAGGAGAAGCAGTTTTCAGATGCTCCCAGGAAGAAGAGGGAGCTGTGGGAGTGCC

1261 TCACCTACCCCCAGCATCCTTTTCATCTCCCCACAGGGCTTTTTGGTCTGGTGAATAATG

1321 CTGGTGTGGCTGGTATCATCGGACCCACACCATGGCTGACCCGGGACGATTTCCAGCGGG

1381 TGCTGAATGTGAACACAATGGGTCCCATCGGGGTCACCCTTGCCCTGCTGCCTCTGCTGC

1441 AGCAAGCCCGGGGCCGGGTGATCAACATCACCAGCGTCCTGGGTCGCCTGGCAGCCAATG

1501 GTGGGGGCTACTGTGTCTCCAAATTTGGCCTGGAGGCCTTCTCTGACAGCCTGAGGTGAG

1561 GGGTACAGGGCTCTGGGTTCCAGGACTAACAGCAGCCCACTCAACAAACGTGGGCCAGCA

1621 GAGGTGGTTAAGATACAGCACATTGGAATAGTTAAGAAGAGACAGTTTAGGGCTAGACTT

1681 CATGGGTTCAATGAAGTCTACCCTTATGTAAGCTTTGTGACCATAAGTAGATTACTTCTC

1741 TTTACCCATTTTTAACGTGtttgttttttgttttttgagatggagtcttgctctgtcgcc

1801 aggctggagtgcagtggcgcgatcttggctcaccacaatttccacccccggggttcaagc

1861 gattctcctgcctcagcctcccgagtagctgggactacaggcatgcgccaccatgcctgg

1921 cttatttttgtatttttagtagagacagggtttcactatgttggccaggttggtctcaaa

1981 ctcctgacctcgtgatccgcccacctcagcctcccaaagtgctgggattacaggtgtgag

2041 ccaccacgcccggccTTGCCTCTCGTCTTTAAACAATAAGGTTCAAAGTTCCGTGGGAGC

2101 ACAAAGGAGACATGATGAGGACAACGGGAGTAGGGCCTGAGtttttttttgttttttttt

2161 ttttaagcgttttgctcttgttgcctaggctggagtgcaatggcgagatctcagctcact

2221 gcaacccctgcctctcaggttcatgtgattctcctgcctcagcctcccgattagctgggc

2281 ttacaggcacgtgccaccactcccagctaatttttttgtatttttagtagagatggagtt

2341 ataccatgttggccaggctggttttgaactcctgacctcaggtgatccacccgacccggc

2401 ctcccaaagtgctgggattacaggcatgagccaccacacacggccCAAGGCCTGAGTTCT

2461 TAGCAGGAGTATAAGGCGCCTAAGCTTAGTCTACCTTCTAAGGAAGCCTGCGTTTGTCAC

2521 CATCACTCAGCAAATAACCTGAATGTCTCCTGTCTCTCAGCCTTAATTTTTCAGGCAGCA

2581 TCATGGGACACATACttttagttttgagacaaggccttgctctcacccagggtggagtgc

2641 agtggtgcagtcacggcccactgaacttcaaactcctaggctcaagcagctcaagcgata

2701 tccgcctcagcctcctgagtagctgagaccacaggcgcgtgccagcatgcctggctagta

2761 tttttttacagatggggtcttgctgtggtgaccagacttgtctccaactcccggcctcaa

2821 gcgatgcttccgcctgggcctcccaaagtgttgggattataggtgtgagccactgcATAC

2881 TGGAACACATACTTTATACTTGAATTTTTTTTTATCCCCTTCCCTCCTGCTCCTTACCTA

2941 TACTTGGATTTCTACATCTGTGCCAGGGCAGTGGGATGTATCCCCACTTTCCCCATCAGC

3001 TTACCCTCCAGCAAATACGAGACTATACCCTTCAATATCCAGCACTCAGGGCTCAACCAT

3061 GTGTTTTGGGAGCAAGGGAATGGGGTTCCTCTAGGTcaggaatcggcaaactcagtactc

3121 aagccagatctggccagctgcctacaagctgataatggttttttttatttttaaatggtt

3181 acattgtaaactgttatataagtacctgataatatcattaattttgtttcttggcctgcc

3241 atgcttaaaatattaactctctggccctttaagaaaaaaacgtgctgacccctgCTCTAG

3301 ATCAAAGAAAACAAACCTCAAAAATACTTTCCTCCCTCTACCCCACTTGACCCTTGTCCC

3361 GGGGCAGTAGGCATCTCCGTCAAAACTCTTGTCCCTGGTCTGTGGTAACTTTCTCAGCTC 2F

3421 CCCAACCCATGTCCCTCAAAGTCCCCTCCCTATAGGGCAAGAACCCAGCAACTTCGCTCT

3481 GCCCCGACTCTAGGCGGGATGTAGCTCATTTTGGGATACGAGTCTCCATCGTGGAGCCTG

3541 GCTTCTTCCGAACCCCTGTGACCAACCTGGAGAGTCTGGAGAAAACCCTGCAGGCCTGCT

3601 GGGCACGGCTGCCTCCTGCCACACAGGCCCACTATGGGGGGGCCTTCCTCACCAAGTGTG

3661 AGTAGCCAGGCCCACACAGGGGCACATGAAGGGAAACAAGTACCAGAAAGGCCAGTCCTG

3721 CATAAGCCTGCTAGGAGGTGGGTGGGGCACCCAGGGCAGGGTTGAGGGTGAACAGGATGT 2R

3781 TACAAGAGTGCCCAGGCCATGTGGAACCTGCCCACTCCCCACACTGAGGAGGGGACTGAG

3841 GGTGACAAGCCCAGGGCCCCAGAAGACAGTACCTAAGATGGGCTGGAGTGAGGAAGGGAA

3901 ACTGATTGCAACCACCTATGGGGCTGCAGACCTGAAAATGCAACAGCGCATCATGAACCT

3961 GATCTGTGACCCGGACCTAACCAAGGTGAGCCGATGCCTGGAGCATGCCCTGACTGCTCG

4021 ACACCCCCGAACCCGCTACAGCCCAGGTTGGGATGCCAAGCTGCTCTGGCTGCCTGCCTC

4081 CTACCTGCCAGCCAGCCTGGTGGATGCTGTGCTCACCTGGGTCCTTCCCAAGCCTGCCCA

4141 AGCAGTCTAC**TGA**ATCCAGCCTTCCAGCAAGAGATTGTTTTTCAAGGACAAGGACTTTGA

4201 TTTATTTCTGCCCCCACCCTGGTACTGCCTGGTGCCTGCCACAAAATAAGCACTAACAAA

4261 AGTGTATTGTTTAAAAAATAAAAAGAAGGTGGGCAGAAATGTGCCCAGTGGAAGGCTGAC

4321 CCCATTTAAGTGCCAACTACTCCAAACCGA
